# The role of kaempferol-induced autophagy on differentiation and mineralization of osteoblastic MC3T3-E1 cells

**DOI:** 10.1186/s12906-016-1320-9

**Published:** 2016-08-31

**Authors:** In-Ryoung Kim, Seong-Eon Kim, Hyun-Su Baek, Bok-Joo Kim, Chul-Hoon Kim, In-Kyo Chung, Bong-Soo Park, Sang-Hun Shin

**Affiliations:** 1 Department of Oral Anatomy, School of Dentistry, Pusan National University, Busandaehak-ro, 49, Mulguem-eup, Yangsan-si, Gyeongsangnam-do 626-870 South Korea; 2 Department of Oral and Maxillofacial Surgery, Pusan National University Dental Hospital, 20, Geumo-ro, Mulgeum-eup, Yangsan-si, Gyeongsangnam-do 626-770 South Korea; 3 Deptment of Oral and Maxillofacial Surgery, Medical center, Dong-A University, 26, Daesingongwon-ro, Seo-gu, Busan, 602-715 South Korea

**Keywords:** Flavonoids, Kaemferol, Osteogenesis, Autophagy

## Abstract

**Background:**

Kaempferol, a kind of flavonol, has been reported to possess various osteogenic biological activities, such as inhibiting bone resorption of osteoclasts and promoting the differentiation and mineralization of preosteoblasts. However, the precise cellular mechanism of action of kaempferol in osteogenesis is elusive.

Autophagy is a major intracellular degradation system, which plays an important role in cell growth, survival, differentiation and homeostasis in mammals. Recent studies showed that autophagy appeared to be involved in the degradation of osteoclasts, osteoblasts and osteocytes, potentially pointing to a new pathogenic mechanism of bone homeostasis and bone marrow disease. The potential correlation between autophagy, osteogenesis and flavonoids is unclear.

**Methods:**

The present study verified that kaempferol promoted osteogenic differentiation and mineralization and that it elevated osteogenic gene expression based on alkaline phosphatase (ALP) activity, alizarin red staining and quantitative PCR. And then we found that kaempferol induced autophagy by acridine orange (AO) and monodansylcadaverine (MDC) staining and autophagy-related protein expression. The correlation between kaempferol-induced autophagy and the osteogenic process was confirmed by the autophagy inhibitor 3-methyladenine (3-MA).

**Results:**

Kaempferol promoted the proliferation, differentiation and mineralization of osteoblasts at a concentration of 10 μM. Kaempferol showed cytotoxic properties at concentrations above 50 μM. Concentrations above 10 μM decreased ALP activity, whereas those up to 10 μM increased ALP activity. Kaempferol at concentrations up to 10 μM also increased the expression of the osteoblast- activated factors RUNX-2, osterix, BMP-2 and collagen I according to RT-PCR analyses. 10 μM or less, the higher of the concentration and over time, kaempferol promoted the activity of osteoblasts. Kaempferol induced autophagy. It also increased the expression of the autophagy-related factors beclin-1, SQSTM1/p62 and the conversion of LC3-II from LC3-I. The application of 3-MA decreased the activity of ALP and the autophagy induced by kaempferol. In the RT-PCR analysis, the expression of RUNX-2, osterix, BMP-2 and collagen I was decreased.

**Conclusion:**

The present study showed that kaempferol stimulated the osteogenic differentiation of cultured osteoblasts by inducing autophagy.

## Background

Bone is a dynamic tissue that is continuously changed by the bone remodeling process [1, 2]. The bone remodeling process involves bone resorption by osteoclasts, followed by bone formation by osteoblasts [3]. Osteoporosis is a well-known bone metabolic disease that occurs due to a decrease in bone mass and density caused by a bone remodeling imbalance [4]. Bone metabolic disorders may result in bone fractures, morbidity and a shortened lifespan [1]. Osteoporosis prevention and bone mass regeneration remain a challenge.

**Fig. 1:**
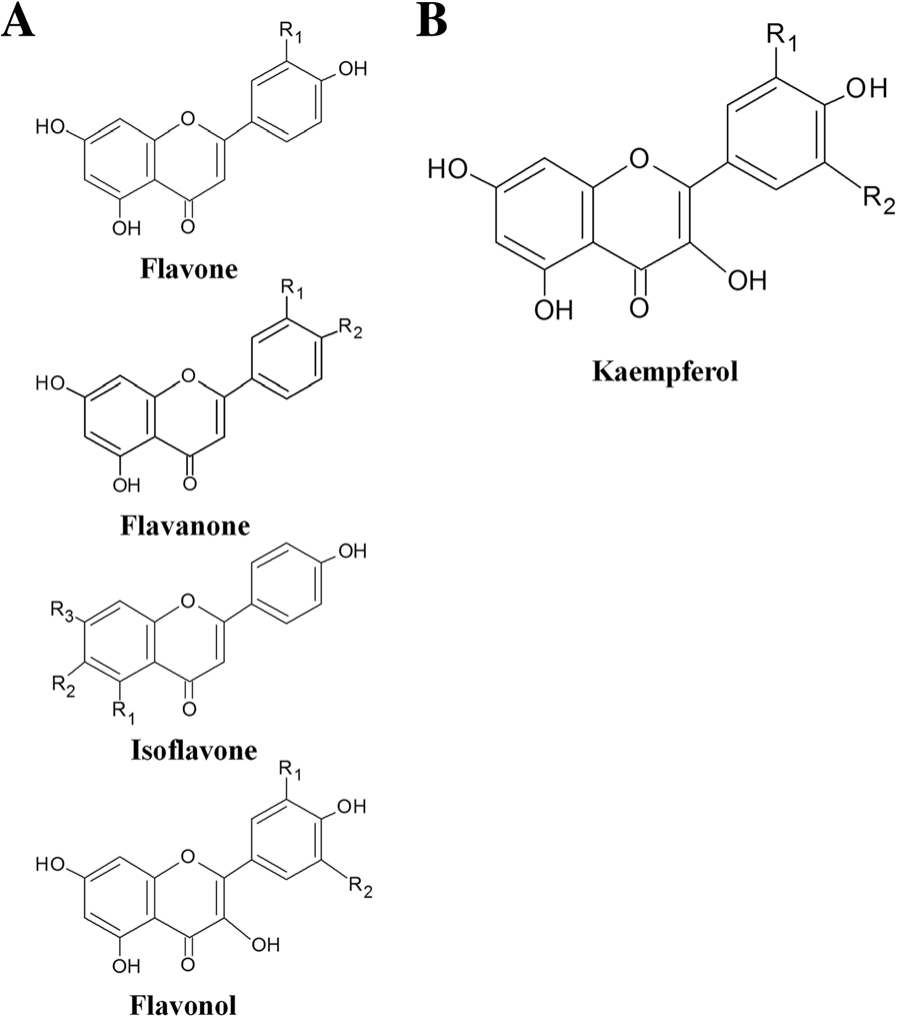
Flavonoid structure. **a** The chemical structure of different flavonoids and (**b**) the chemical structure of kaempferol

Flavonoids, small polyphenolic molecules commonly found in roots, leaves and fruits of dietary plants, have well-known antioxidant, anti-inflammatory, anticancer and antibacterial activities [5–7]. All flavonoids are composed of two phenolic rings, which are connected by a 3-carbon unit. They are classified as flavonols, flavones, flavanones, isoflavones, catechins, anthocyanidins and chalcones according to the hydroxylation and oxidation binding positions of their chemical structures (Fig. 1) [8, 9]. Several recent studies have reported that flavonoids, such as genistein, icariin, quercetin, taxifolin and kaempferol have osteogenic, antiosteoclastogenic and antiadipogenic effects [10–13].

Kaempferol, a kind of flavonol, is derived from the rhizome of *Kaempferia galanga L*., which is used in traditional medicine in Asia for hypertension, abdominal pain, headaches and rheumatism [14, 15]. Kaempferol has been reported to possess various osteogenic biological activities, such as inhibiting bone resorption of osteoclasts [15] and promoting the differentiation and mineralization of preosteoblasts [16, 17]. However, the precise cellular mechanism of action of kaempferol in osteogenesis is elusive.

Autophagy is a major intracellular degradation system, which plays an important role in cell growth, survival, differentiation and homeostasis in mammals [18]. Various stimuli induce autophagy, including stress, cytokines, pathogens, protein aggregation and damaged or surplus organelles, which are recycled to produce energy for cellular repair and homeostasis [19]. Recent studies showed that autophagy appeared to be involved in the degradation of osteoclasts, osteoblasts and osteocytes, potentially pointing to a new pathogenic mechanism of bone homeostasis and bone marrow disease [20].

The potential correlation between autophagy, osteogenesis and flavonoids is unclear. The aim of this study was that kaempferol promoted both bone formation and autophagy and that kaempferol-induced autophagy played a major role in controlling the osteogenic process in MC3T3-E1 osteoblastic cells.

## Methods

### Reagents

The reagents kaempferol, 3-[4,5-dimethylthiazol-2-yl]2,5-diphenyl tetrazolium bromide (MTT), MDC, and AO, were purchased from Sigma (St. Louis, MO, USA). The reagent 3-MA (a type-III phosphatidylinositol 3-kinase [PI-3K] inhibitor) was obtained from Calbiochem (La Jolla, CA, USA). Antibodies against beclin-1 and ATG5 were purchased from Cell Signaling Technology (Beverly, MA, USA). Antibodies against LC3 (Sigma, Louis, MO, USA) were also used. SQSTM1/p62, β-actin antibody, mouse anti-rabbit IgG antibody and rabbit antimouse IgG antibodies were purchased from Santa Cruz Biotechnology (Santa Cruz, CA, USA). All other chemicals and reagents were purchased from Sigma, unless otherwise specified.

### Cell culture

MC3T3-E1 cells, a clonal mouse osteoblastic cell line, were purchased from ATCC (Rockville, MD, USA). The cells were cultured in alpha-modified Eagle’s medium (α-MEM), without ascorbic acid (Gibco BRL, Gland Island, NY, USA), with 10 % fetal bovine serum (FBS) and 1 % penicillin-streptomycin at 37 °C. To induce differentiation, the cells were seeded onto a 24-well culture plate and allowed to reach confluence. At confluence (day 0), the cells were transferred to α-MEM containing 10 % FBS, 1 % penicillin-streptomycin, 10 mM of β-glycerophosphate, and 100 μg/ml of ascorbic acid and then incubated for different intervals (3, 7, and 14 days) in a humidified atmosphere of CO_2_ at 37 °C.

### Treatment with kaempferol

Stock solutions of kaempferol (100 mM) were made by dissolving them in DMSO, followed by freezing at −20 °C until use. The stock was diluted to their concentration with α-MEM when needed. Prior to the kaempferol treatment, the cells were grown to about 80–100 % confluence and then exposed to kaempferol at different concentrations (0.1–1000 μM) for at least 24 h, up to 14 days. Cells grown in medium containing an equivalent amount of DMSO without kaempferol served as a control. For autophagy control, the cells were grown in Earle’s balanced salt solution (EBSS).

### MTT assay

Cell proliferation was performed using an MTT assay according to the manufacturer’s instructions. The cells (1 × 10^4^) were cultured in a 96-well plate and incubated for 24 h. They were treated with kaempferol at various concentrations (0.1–1000 μM) for 24–72 h. After the treatment, 100 μl of MTT (final concentration of 0.5 mg/ml) were added and incubated for an additional 4 h at 37 °C to induce the production of formazan crystals, and the supernatants were discarded. The medium was aspirated, and formazan crystals that formed were dissolved in DMSO. Cell viability was monitored on an ELISA reader (Sunrise Remote Control, Tecan, Austria) at an excitation emission wavelength of 570 nm.

### Measurement of alkaline phosphatase (ALP) activity

To investigate osteogenic differentiation, the cells were treated with 10 μM of kaempferol, and the ALP activity was determined 3 and 7 days later. The cells were plated in 6-well plates at a density of 2 × 10^5^ cells/well. The protein fraction protocol was using previous method [21]. The ALP activity in the cellular fraction was measured with an Alkaline Phosphatase Assay Kit (Abcam, Cambridge, MA, USA). A standard curve was created using p-nitrophenol as a standard, and each value was normalized to the protein concentration. The ALP activity of each sample was normalized by the protein concentration and measured by ELISA reader at 405 nm.

### Alizarin red staining

The day 7 and 14 samples were fixed in 4 % paraformaldehyde for 15 min. To detect calcium deposition, the cells were stained with 2 % alizarin red solution (pH 4.2) for 10 min and washed twice with distilled water. For quantification, the cells were destained with 10 % w/v cetylpyridinium chloride for 30 min at room temperature and transferred to a 96-well plate, and the absorbance was measured at 550 nm using an ELISA reader.

### Acridine orange (AO) and monodansycadaverine (MDC) staining

The cells were grown on coverslips and treated with kaempferol. After 24 h, they were stained with 50 μM of MDC, a selective fluorescent marker for autophagic vacuoles [22], at 37 °C for 30 min. The cellular fluorescence changes were observed using a fluorescence microscope (Axioskop, Carl Zeiss, Germany). As an autophagy control, the cells were starved using EBSS. Autophagy is characterized by acidic vesicles formation as described previously [23]. For further detection of the acidic cellular compartment, AO was used. AO emits bright red fluorescence in acidic vesicles but fluoresces green in the cytoplasm and nucleus [23]. The cells were stained with 0.1 μg/ml of AO for 15 min and washed with PBS. The formation of acidic vesicular organelles (AVOs) was evaluated under a confocal microscope LSM 700 (Carl Zeiss, Germany).

### Western blot assay

The cells were plated at a density of 2 × 10^6^ cells in 100 mm culture dishes. They were washed twice with ice-cold PBS and centrifuged at 2,000 rpm for 10 min. The total cell proteins were lysed with an RIPA buffer (300 mM NaCl, 50 mM Tris-HCl [pH 7.6], 0.5 % TritonX-100, 2 mM PMSF, 2 μg/ml aprotinin, and 2 μg/ml leupeptin) and incubated at 4 °C for 1 h. The lysates were centrifuged at 14,000 revolutions per min for 15 min at 4 °C, and sodium dodecyl sulfate (SDS) and sodium deoxycholic acid (final concentration of 0.2 %) were added. The protein concentrations of the cell lysates were determined with a Bradford protein assay (Bio-Rad, Richmond, CA, USA), and BSA was used as a protein standard. A sample of 20 μg of protein in each well was separated and loaded onto 7.5–15 % SDS/PAGE gels. The gels were transferred to PVDF (Amersham GE Healthcare, Little Chalfont, UK) and reacted with each antibody. Immunostaining with antibodies was performed with a SuperSignal West Femto-enhanced chemiluminescence substrate and detected with an Alpha Imager HP (Alpha Innotech, Santa Clara, USA). Equivalent protein loading was confirmed by Ponceau S staining.

### Real-time PCR

Total RNA was removed from the cultured MC3T3-E1 cells using Trizol reagent (Invitrogen, Carlsbad, NM, USA) according to the manufacturer’s instructions. Total RNA (2 μg) was reverse-transcribed using the RevertAid First-Stand Synthesis System kit for the real-time polymerase chain reaction (Thermo Fisher Scientific, Pittsburgh, PA, USA) according to the manufacturer’s protocol. Real-time PCR was performed on an ABI 7500 Fast Real-Time PCR System (Applied Biosystems 7500 System Sequence Detection System, software version 2.6.1) using SYBR Green PCR Master Mix (Applied Biosystems, Foster City, CA, USA). The running conditions were as follows: The temperature profile of the reaction was 95 °C for 15 min, followed by 40 cycles of denaturation at 95 °C for 30 s, annealing at 58 °C for 30 s, and extension at 72 °C for 60 s. The primers used in this study are shown in Table 1.

**Table 1:** Primers used in this study

Gene	Sense (5′-3′)	Antisense (5′-3′)
RUNX-2	CCCAGCCACCTTTACCTACA	TATGGAGTGCTGCTGGTCTG
Osterix	GCAAGAGGTTCACTCGCTCT	GTGGTCGCTTCTGGTAAAGC
LC3	TTCTTCCTCCTGGTGAATGG	ATTGCTGTCCCGAATGTCTC
SQSTM1/p62	GAAGCTGCCCTATACCCACA	GGTCTGTAGGAGCCTGGTGA
GAPDH	AACTTTGGCATTGTGGAAGG	GGATGCAGGGATGATGTTCT

### Statistical analysis

All the experiments were performed in triplicate, and the results were expressed as the mean ± SD. Statistical analyses were performed with the SPSS 13.0 statistical software program (SPSS Inc., IL, USA). A one-way ANOVA was used for multiple comparisons in the statistical analysis.

## Results

### Kaempferol did not exert a cytotoxic effect below 50 μM in the MC3T3-E1 cells

**Fig. 2:**
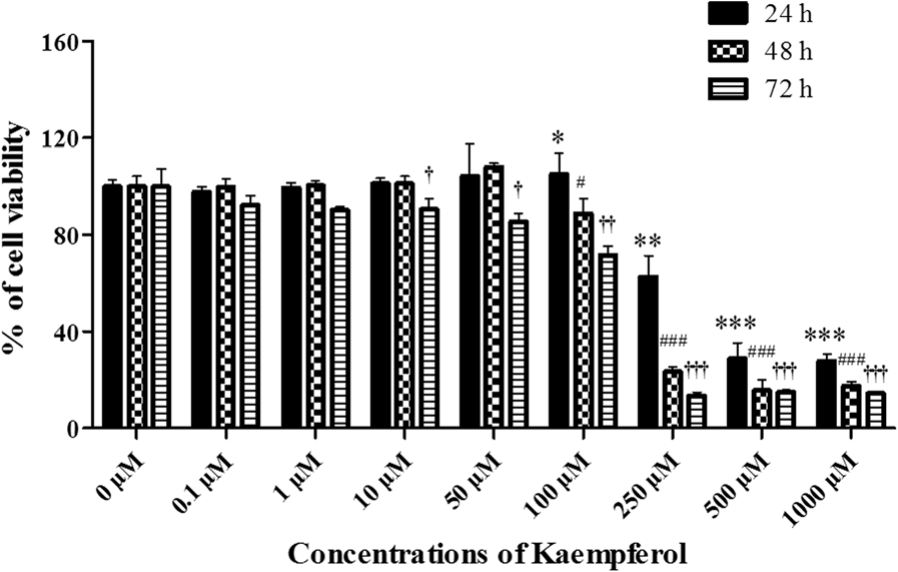
Cytotoxic effect of kaempferol on the MC3T3-E1 cells. The cytotoxicity of various concentrations of kaempferol to MC3T3-E1 cells exposed to the treatment for 24–72 h was determined with an MTT assay. Concentrations of 0.1–10 μM had no obvious effect on the cells. However, concentrations over 10 μM significantly reduced the viability of the MC3T3-E1 cells compared with untreated controls. The data are expressed as the mean ± SD (*n*=6). The statistical significance of each group was analyzed by a one-way ANOVA and Tukey’s HSD test (*p < 0.05, **p < 0.01, ***p < 0.001 at 24 h, #*p* < 0.05, ##*p* < 0.01, ###*p* < 0.001 at 48 h, and †*p* < 0.05, ††*p* < 0.01, †††*p* < 0.001 at 72 h for the difference between the treatment group (0.1–1000 μM) compared to the non-treated group (0 μM))

We first examined the cytotoxicity of kaempferol over a wide concentration range using an MTT assay in the MC3T3-E1 cells. The cells were treated with kaempferol (0.1–1000 μM) and incubated for 24–72 h. At 0.1–50 μM, the kaempferol treatment had no cytotoxic effect. However, over 50 μM, it potently reduced the viability of the MC3T3-E1 cells in a dose-dependent manner. Low concentrations of kaempferol (less than 50 μM) did not have a significant effect on the viability or survival of the MC3T3-E1 cells (Fig. 2) or on the induction of differentiation, mineralization, or autophagy in later experiments. Thus, we used a concentration of kaempferol below 50 μM in subsequent experiments.

### Kaempferol induced the differentiation, mineralization of osteoblasts and the markers gene expression of osteoblasts in the MC3T3-E1 cells

**Fig. 3:**
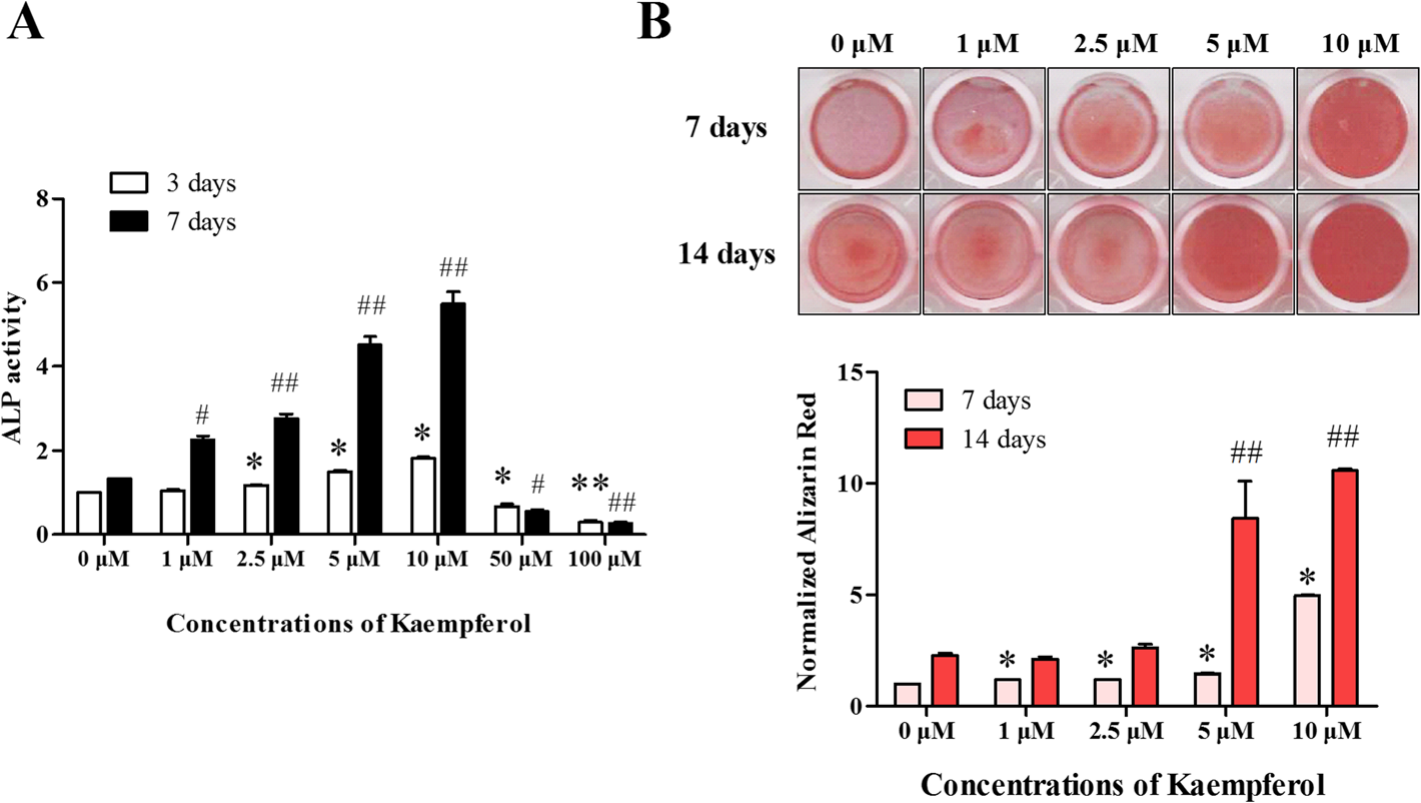
Effect of kaempferol on osteoblast differentiation and mineralization. **a** The kaempferol treatment of the cultured MC3T3-E1 cells increased ALP activity in a dose- (1–100 μM) and time-dependent (3 and 7 days) manner. The data are expressed as the mean ± SD (*n*=6). The statistical significance of each group was analyzed by a one-way ANOVA and Tukey’s honestly significant difference (HSD) test (*p < 0.001, 0 μM [3 days], #*p* < 0.01, 0 μM [7 days], and ##*p* < 0.001, 0 μM [7 days] for the difference between the control and treatment groups). **b** The addition of kaempferol (1–10 μM) induced mineralization of the cultured osteoblasts. After 7 and 14 days of the kaempferol treatment, nodules were detected, and these increased in a dose-dependent manner, as shown by alizarin red staining. The data are expressed as the mean ± SD (*n*=6). The statistical significance of each group was analyzed by a one-way ANOVA and Tukey’s HSD test (*p < 0.001 at 7 days and ##*p* < 0.001 at 14 days for the difference between the control and treatment groups)

To determine the concentration of kaempferol that controlled the differentiation and mineralization of osteoblasts, we performed an ALP activity assay and alizarin red staining. The ALP activity was used as an initial indicator of osteoblast differentiation. ALP activity was decreased at a concentration above 10 μM. To evaluate the ALP activity, the cells were treated with various concentrations of kaempferol (1, 2.5, 5, 10, 50 and 100 μM) and incubated for 3 to 7 days. As shown in Fig. 3a, ALP activity increased significantly in accordance with the increase in the concentration of kaempferol after 7 days. The level of ALP activity of was particularly high at a kaempferol concentration of 10 μM, but the effect decreased after 3 days of the treatment.

Osteoblasts give rise to bone formation through a process of cell proliferation, differentiation and mineralization. During mineralization, they produce extracellular calcium deposits, which can be detected by alizarin red staining. The kaempferol-treated cells were incubated for 7–14 days and then stained and quantified using alizarin red and an ELISA reader. After 7 days, kaempferol at a concentration of 10 μM induced calcified nodules that stained a deep red color compared to the control and other concentrations. The cells were stained more deeply after 14 days of the kaempferol treatment than after 7 days, and the intensity of the alizarin red was also greater (Fig. 3b). These results provide convincing evidence that kaempferol efficiently induces the differentiation and mineralization of MC3T3-E1 cells at a concentration of 10 μM.

**Fig. 4:**
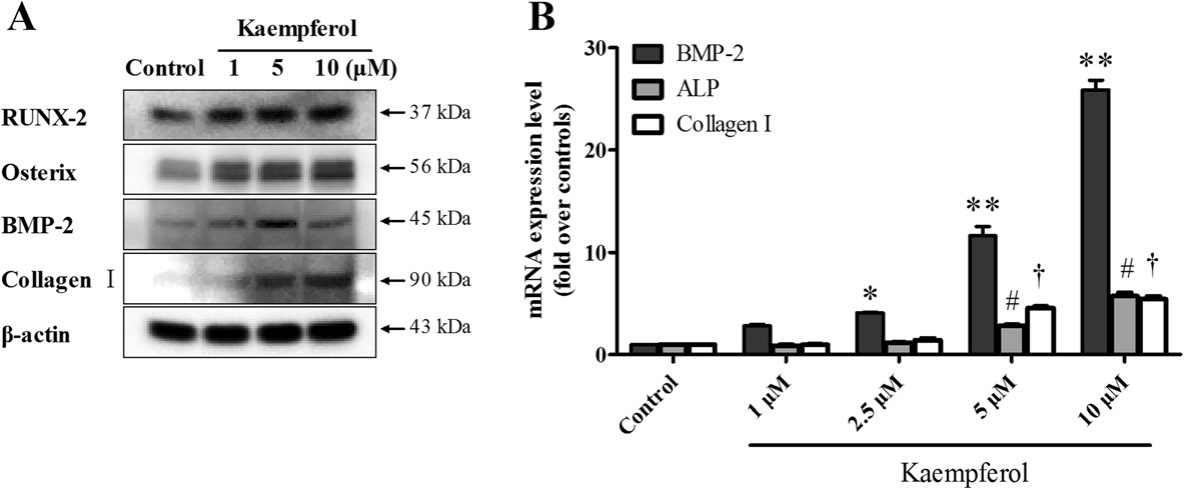
Effect of kaempferol on osteogenesis-associated factors. **a** The cells were treated with kaempferol (1–10 μM) for 24 h. Western blotting showed that kaempferol activated the osteogenic proteins such as RUNX-2, osterix, BMP-2 and collagen I. β-actin was used as the internal control. **b** The total RNA was extracted from the cultures to perform quantitative PCR. The mRNA expression of osteogenesis-associated genes, including BMP-2, ALP and collagen I, increased in a dose-dependent manner. The data are expressed as the mean ± SD (*n*=6). The statistical significance of each group was analyzed by a one-way ANOVA and Tukey’s HSD test (*p < 0.05 [BMP-2], **p < 0.001 [BMP2], #*p* < 0.001 [ALP], and †*p* < 0.001 [collagen I] for the difference between the treatment group compared to the control group)

The effect of kaempferol on osteoblast differentiation was further confirmed with Western blotting and real-time PCR. After 24 h of the kaempferol treatment, the total protein and RNA were isolated, and the expression of the osteoblast differentiation makers, RUNX-2, osterix, BMP-2 and collagen I, was analyzed. The protein expression levels of all the genes increased in a dose-dependent manner in response to the kaempferol treatment. The mRNA levels showed dramatic results more than Western blotting, especially, BMP-2 expression level increases remarkably at 5–10 μM of kaempferol (Fig. 4a, b). As kaempferol significantly increased ALP activity, the intensity of alizarin red, and the mRNA and/or protein levels of the osteogenic markers, we elucidate that kaempferol induced osteoblastic differentiation in the MC3T3-E1 cells and that it has osteogenic potential.

### Kaempferol induced autophagy

**Fig. 5:**
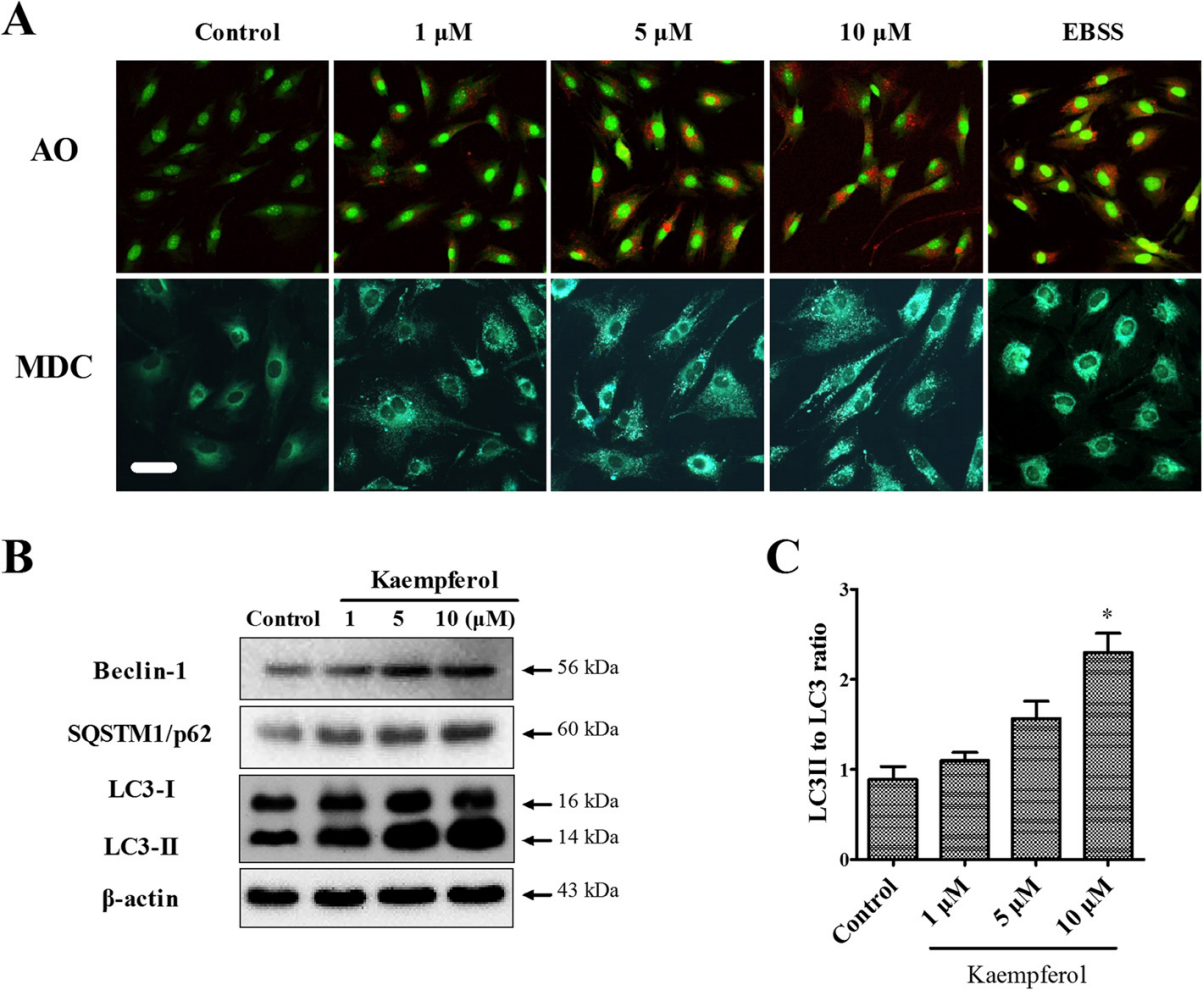
Induction of autophagy in the kaempferol-treated MC3T3-E1 cells. **a** The cells were grown on coverslips and treated with various concentrations of kaempferol (1–10 μM) for 24 h. The formation of autophagosomes and autolysosomes was analyzed by fluorescence microscopy. The upper and lower panels show images of AO and MDC staining, respectively, captured using a fluorescence microscope. Scale bar, 50 μm. **b** Western blotting used antibodies specific for beclin-1, SQSTM1/p62, LC3 and β-actin. β-actin was used as an internal control. The results shown are representative of at least three replicates. **c** After kaemferol treatment, the LC3-II to LC3-I ratio is increased in dose-dependently

We next investigated whether autophagy occurred in the kaempferol-treated MC3T3-E1 cells. We confirmed the formation of autophagic vacuoles in response to kaempferol by staining with AO and MDC. Autophagy is usually characterized by increased formation of AVOs, which represent lysosomes and autophagolysosomes. AVOs appear as red fluorescent and punctate by AO and MDC. As shown in the upper panel of Fig. 5a, red-colored autophagic vacuoles were observed following the treatment with 1–10 μM of kaempferol for 24 h. The formation of MDC-labeled vacuoles was also observed in the kaempferol-treated MC3T3-E1 cells but not in the control cells (Fig. 5a, lower panel). EBSS was used as a positive control of autophagy. The results of the MDC and AO staining of autophagic vacuoles indicated that the kaempferol treatment of the MC3T3-E1 cells was sufficient to instigate an autophagic response. To identify kaempferol-induced autophagy in the MC3T3-E1 cells, we conducted a Western blot assay to detect various autophagy markers, such as beclin-1, SQSTM1/p62 and LC3. SQSTM1/p62 and beclin-1, which are activated by autophagy, accumulated in a dose-dependent manner in the kaempferol-treated MC3T3-E1 cells. The conversion rates of LC3-I to LC3-II, as well as the total levels of LC3 proteins, increased in the kaempferol-treated MC3T3-E1 cells in a dose-dependent manner. During autophagy, LC3-I was converted to LC3-II, it is the most representative and important event in the autophagic process. The kaempferol-induced conversion of LC3-I to LC3-II demonstrates that this treatment can induce autophagy, as shown in the previous experiments (Fig. 5b, c).

### Kaempferol-induced autophagy was associated with the differentiation of osteoblasts

**Fig. 6:**
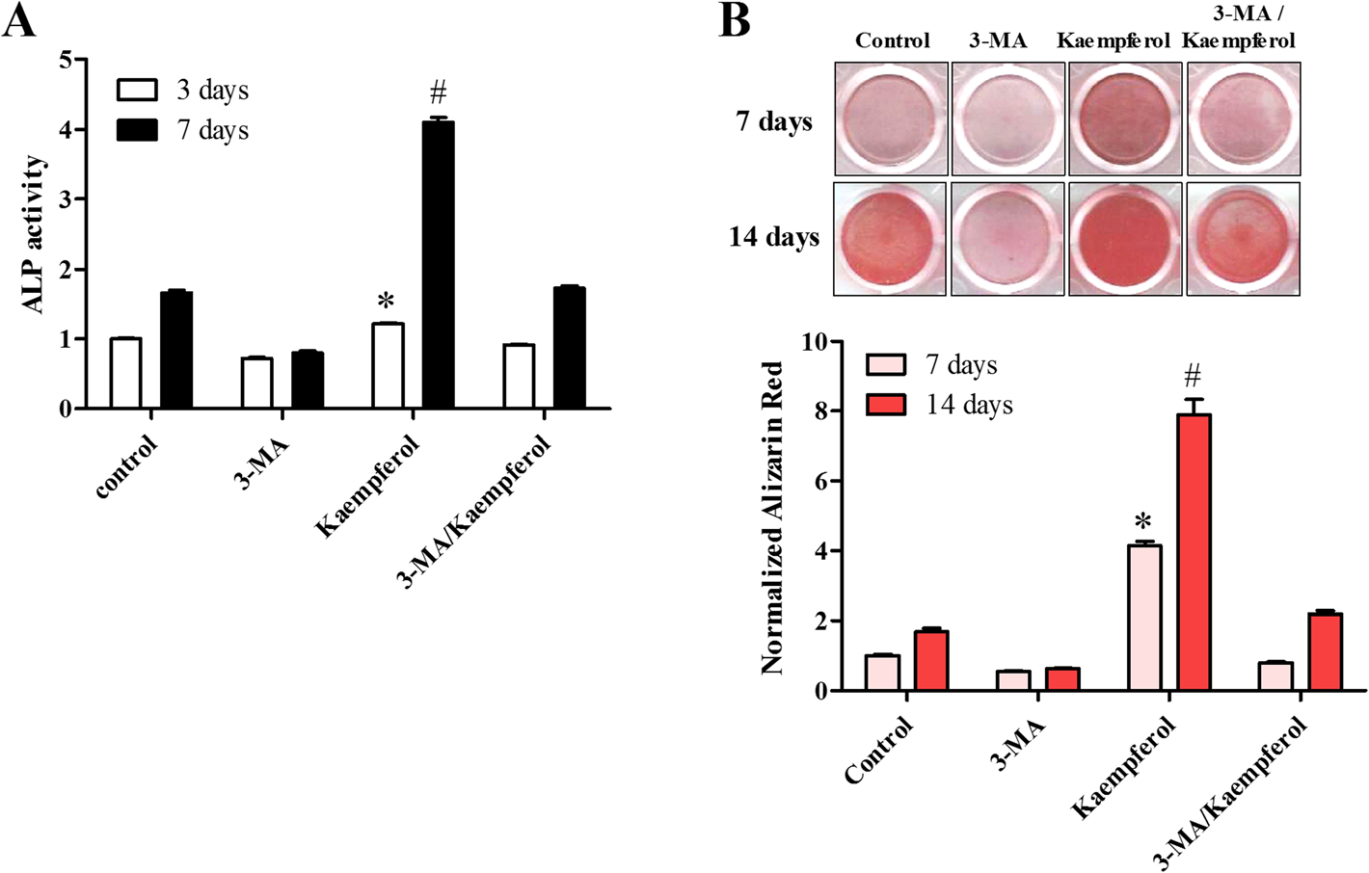
Effect of 3-MA on the differentiation and mineralization of osteoblasts by kaempferol. **a** The MC3T3-E1 cells were pretreated with an autophagy inhibitor, 3-MA (1 mM), for 1 h and then treated with 10 μM of kaempferol. ALP activity showed that 3-MA blocked the differentiation of the MC3T3-E1 cells induced by the kaempferol (10 μM) treatment. The data are expressed as the mean ± SD (*n*=6). The statistical significance of each group was analyzed by a one-way ANOVA and Tukey’s HSD test (*p < 0.001 [3 days] and #*p* < 0.001 [7 days] for the difference between the control compared to the treatment group). **b** 3-MA also inhibited osteoblast mineralization induced by the addition of kaempferol, as shown by alizarin red staining. The data are expressed as the mean ± SD (*n*=6). The statistical significance of each group was analyzed by a one-way ANOVA and Tukey’s HSD test (*p < 0.001 [7 days] and #*p* < 0.001 [14 days] for the difference between the control group compared to the treatment groups)

**Fig. 7:**
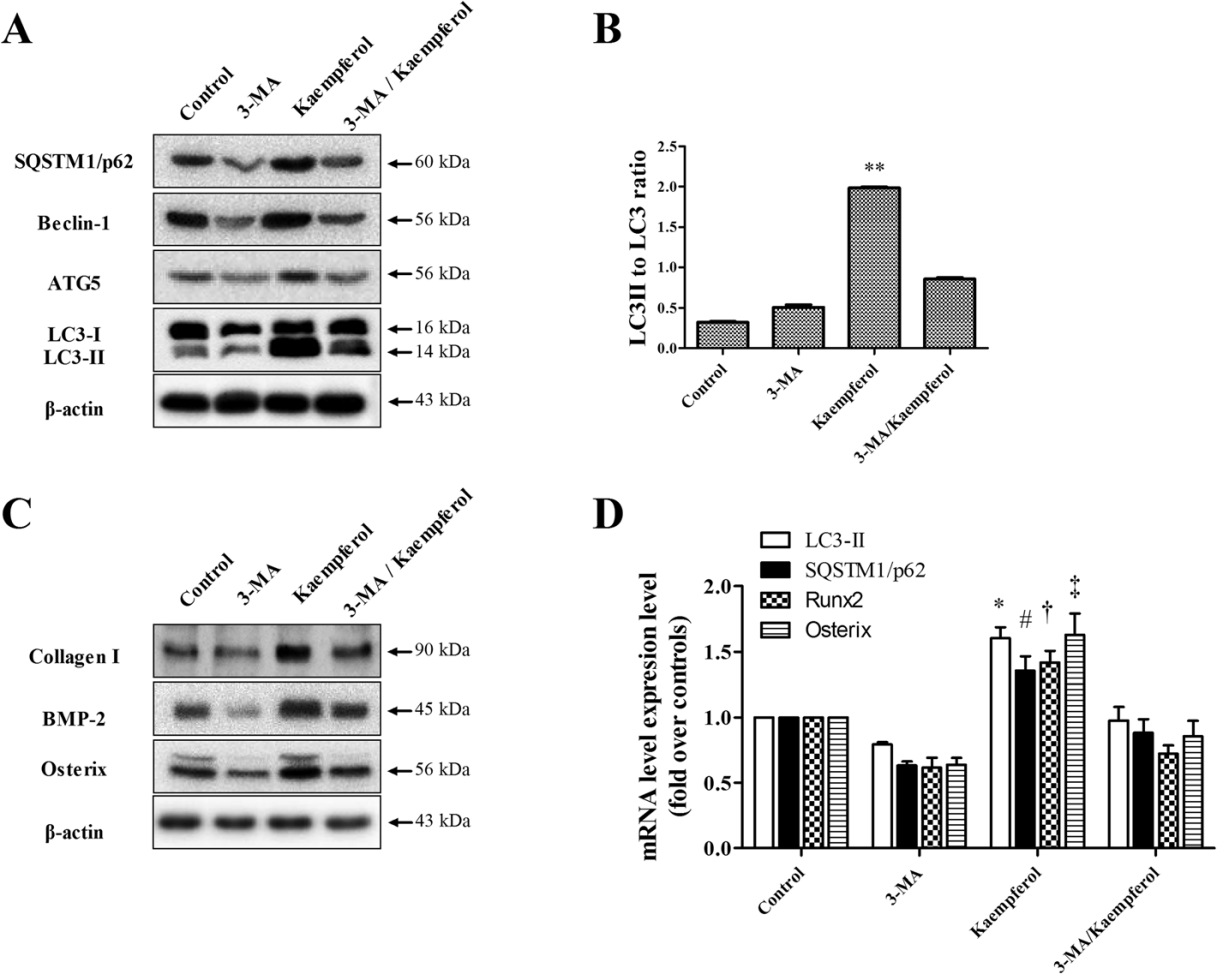
Effect of kaempferol on osteogenic factors associated with autophagy. The relationship between kaempferol-induced autophagy and osteogenic differentiation was confirmed via 3-MA, Western blotting (**a**, **b**, **c**) and real-time PCR (**d**). 3-MA clearly inhibited both autophagy and the osteogenic factors activated by kaempferol. The data are expressed as the mean ± SD (*n*=6). The statistical significance of each group was analyzed by a one-way ANOVA and Tukey’s HSD test (*p < 0.01 [LC3-II], #*p* < 0.01 [SQSTM1/p62], †*p* < 0.01 [Runx2] and ‡*p* < 0.01 [Osterix] for the difference between the control compared to the treatment groups)

Finally, we hypothesized that kaempferol-induced autophagy was associated with osteoblast differentiation and bone formation. To test this hypothesis, we used 3‐MA, which prevents autophagy by blocking autophagosome formation via the inhibition of the type III phosphatidylinositol 3-kinase (PI-3K), to determine whether kaempferol-induced autophagy played an important role in the differentiation of the MC3T3-E1 cells. We found that 3-MA blocked the kaempferol-induced osteogenic differentiation and mineralization, as confirmed by a reduction in ALP activity and alizarin red staining (Fig. 6a, b). In addition, as shown in Fig. 7a, b and c, kaempferol activated autophagy-related factors, including SQSTM1/p62, beclin-1, ATG5 and LC3, but their induction was inhibited by 3-MA. This result clearly confirms that kaempferol is associated with autophagy. 3-MA also blocked the induction of osteoblast differentiation markers, such as collagen I, BMP-2 and osterix, in addition to the expression of RUNX-2 and osterix mRNA levels induced by the kaempferol treatment in the MC3T3-E1 cells (Fig. 7d). These results clearly support the hypothesis that kaempferol induces autophagy and that autophagy activates osteoblast differentiation.

## Discussion

Flavonoids are naturally occurring compounds found in food in the human diet [6]. They are also used in medicine [6]. Flavonoids are known to be very effective in the prevention of cancer and cardiovascular, neurodegenerative [24] and metabolic bone diseases [25]. Numerous recent studies reported that flavonoids controlled osteoblast and osteoclast functions [13, 17]. They showed that kaempferol, in particular, exerted a potent inhibitory effect on the bone resorbing activity of osteoclasts [15] and that it promoted osteoblastic differentiation and mineralization of an osteoblastic cell line [16]. Autophagy has well-known physiological and pathological roles in many organs [26, 27]. However, the physiological roles of autophagy in bone homeostasis and metabolic bone disease are largely unknown [28]. Also studies of kaempferol have shown that it has anti-inflammatory [29], breast cancer prevention [30] and neuroprotection properties [31]. MC3T3-E1 cells have been used to investigate the modulatory effects of various flavonoids on osteogenic characteristics. These cells provide a useful model for studying bone cell proliferation and differentiation and a renewable culture system to determine the molecular mechanism of osteoblast maturation and the formation of the bone-like extracellular matrix [32]. Therefore, in the present study, we used MC3T3-E1 osteoblastic cells to determine the osteogenesis and autophagy potential of kaempferol.

We first determined the concentration of kaempferol that promoted osteoblast function by assessing the mineralization of MC3T3-E1 cells. Kaempferol showed cytotoxicity at concentrations greater than 10 μM. Recently, Podder et al. [33] reported that kaempferol at concentrations up to 5 μM had no cytotoxic effects but that it suppressed the MUC5AC gene, resulting in a chronic cough and excessive sputum production. They also reported that the treatment had a cyto-protection effect in bronchial epithelium BEAS-2B cells. Another study found that kaempferol (10 μM) activated the transcriptional activity and induction of osteoblast differentiation biomarkers, including ALP, and that it promoted the mineralization of rat osteoblasts [34]. Increasing differentiation and mineralization of osteoblasts is the ultimate aim of all bone anabolic therapy [35]. The activity of ALP is known to be increased in osteoblasts in the early phase of differentiation. ALP is an essential indicator of the mineralization capacity, and it is expressed by all osteoblastic cells. ALP activity was also reported to be a characteristic of osteogenic cells in culture and to promote bone matrix mineralization [36]. We observed that kaempferol stimulated ALP activity and the formation of mineralized nodules at a maximum concentration of 10 μM in the MC3T3-E1 cells, thereby suggesting a bone anabolic action for kaempferol in osteoblasts. The transcription of genes for several bone differentiation markers (collagen I, RUNX-2, osterix and BMP-2) was up-regulated in response to the kaempferol treatment in cultured osteoblasts. In all cases, the transcripts encoding these markers were significantly induced (two to three-fold) by kaempferol at a concentration of 10 μM. At this concentration, the level of BMP-2 increased 25-fold.

During autophagy, cytoplasmic materials are enclosed in a double‐membrane structure called an autophagosome, which fuses with lysosomes to form autolysosomes, where degradation of the cytoplasmic materials occurs [28]. In addition to the role of autophagy in adaptive responses to starvation, quality control of intracellular proteins and organelles, antiaging, suppression of tumor formation, elimination of intracellular microbes, and antigen presentation, there is increasing evidence that it plays important roles in differentiation and development [28]. The essential proteins of autophagy involved in autophagosome formation, ATG5, beclin-1 and LC3, that lead to early lethality, prevent us from studying the physiological role of autophagy in bone development [37]. The results of the present study provide clear evidence for a positive role of autophagy in bone development and osteoblast differentiation. Although ATG5 and SQSTM1/p62 have been shown to regulate autophagic degradation [38], their importance in bone cell activity is not yet known [20]. Recently, Ha et al demonstrated that bioactive silica-based nanoparticle formulation stimulates osteoblast differentiation and autophagy including the increase in LC3β-II and SQSTM1/p62, but its interaction has yet to be demonstrated [19]. The conversion of cytosolic LC3-I to autophagic vesicle-associated phospholipid conjugate from LC3-II has been widely used to monitor autophagy flux [31]. The lipidated form of LC3 transformed from LC3-I to LC3-II is considered to be an autophagosomal marker due to its localization and aggregation on autophagosomes [32, 33]. Our data clearly showed that kaempferol activated autophagic-related genes, such as ATG5, beclin-1, SQSTM1/p62 and LC3, and that it induced autophagosome formation. These findings suggest that kaempferol augments autophagy in osteoblasts.

Given that 3‐MA might have effects on lysosomes that are independent of autophagy, we used an alternate approach to block the formation of autophagosomes (i.e., knocking down the expression of key autophagy genes, using either ATG5 or beclin-1 specific small interfering RNA) [39]. While early autophagy inhibitor 3-MA inhibited the conversion of LC3-I to LC3-II, we found that 3-MA reduced both osteoblast differentiation and the expression of osteogenic gene makers, indicating that kaempferol induced autophagy and that autophagy promoted osteoblast differentiation of the MC3T3-E1 cells.

The present study, for the first time, demonstrate the role of kaempferol in osteoblast differentiation and autophagy. Methodological innovations in molecular biological studies and new animal models will help to shed light on this issue. The findings of the current study may help to delineate the potential role of kaempferol in the treatment of bone metabolism disorders.

## Conclusion

The present study showed that kaempferol stimulated the osteogenic differentiation of cultured osteoblasts by inducing autophagy.

## Abbreviations

3-MA: 3-methyladenine
ALP: Alkaline phosphatase
ANOVA: Analysis of variance
AO: Acridine orange
ATCC: American type culture collection
AVOs: Acidic vesicular organelles
DMSO: Dimethyl sulfoxide
EBSS: Earle’s balanced salt solution
MDC: Monodansylcadaverine
MTT: Methyl thiazolyl tetrazolium
PI-3K: Phosphatidylinositol 3-Kinase
PVDF: Polychlorotrifluoroethylene
SDS: sodium dodecyl sulfate
